# Cross-Cultural Validation of the English Chronic Pain Myth Scale in Emergency Nurses

**DOI:** 10.1155/2019/1926987

**Published:** 2019-03-14

**Authors:** Geraldine Martorella, Anaïs Lacasse, Michelle Kostic, Glenna Schluck

**Affiliations:** ^1^TMH Center for Research and Evidence-Based Practice, College of Nursing, Florida State University, 104F Vivian M. Duxbury Hall, 98 Varsity Way, Tallahassee, FL 32306, USA; ^2^Laboratoire de Recherche en Épidémiologie de la Douleur Chronique, Département des Sciences de la Santé, Université du Québec en Abitibi-Témiscamingue, 445 Boul. de l'Université, Rouyn-Noranda (Qc), Canada J9X 5E4; ^3^College of Nursing, Florida State University, Tallahassee, FL, USA

## Abstract

**Background:**

Utilization of the emergency department (ED) by patients seeking relief from chronic pain (CP) has increased. These patients often face stigmatization, and the ED is no exception. The French-Canadian Chronic Pain Myth Scale (CPMS) was developed to evaluate common societal misconceptions about CP including among healthcare providers. To our knowledge, no tool of this nature is available in English.

**Objectives:**

This study thus aimed at determining to what extent a new English adaptation of the CPMS could provide valid scores among US emergency nurses. The internal consistency, construct validity, and internal structure of the translated scale were thus examined.

**Methods:**

After careful translation of the scale, the English CPMS was administered to 482 emergency nurses and its validity was explored through a web-based cross-sectional study.

**Results:**

Acceptable reliability (*α* > 0.7) was reported for the first and third subscales. The second subscale's reliability coefficient was below the cutoff (*α*=0.67) but is still considered adequate. As expected, statistically significant differences were found between nurses suffering from CP vs nurses not suffering from CP, supporting the construct validity of the scale. After exploratory factor analysis, similar internal structure was found supporting the 3-factorial nature of the original CPMS.

**Conclusion:**

Our results provide support for the preliminary validity of the English CPMS to measure knowledge, beliefs, and attitudes towards CP among emergency nurses in the United States.

## 1. Introduction

Approximately 100 million of adult Americans suffer from chronic pain (CP), i.e., pain present daily or repeatedly for more than three months, with an estimated national economic cost of 560–635 billions annually [1]. In the United States (US), it is generally recognized that access to care for CP patients is particularly scarce [2, 3]. While waiting, patients often get discouraged and need to find some relief. Visiting the emergency department (ED) becomes their sole alternative [4, 5]. Utilization of the ED by patients seeking relief from CP has increased and is estimated to be up to 16% of all ED visits [6, 7]. In a fast-paced environment such as the ED, patients with CP are challenging to healthcare providers (HCP) [5, 8]. In fact, repercussions of CP such as depressive symptoms and pain interference with activities are not properly addressed [5, 9]. Although the ED is not the best clinical environment to assess and treat these patients, CP is more and more prevalent and HCP in acute care settings have to adjust to this new clinical reality and offer some support to these patients [4, 5, 8].

The Institute of Medicine identified CP as a care priority in the US and highlighted the need to delineate barriers and strategies to improve pain management practice [1]. One of the main barriers remains stigmatization of people living with CP by the community, including HCP [10, 11]. Unfortunately, HCP in the ED are no exception [5, 8, 12]. Several scales have been developed to assess knowledge, beliefs, and attitudes of HCP towards pain and its treatment [13–20], but they are specific to a particular expertise (e.g., diagnosis and prescription) or a pain condition (e.g., back pain) and seem to occult some of the common misbeliefs and myths towards CP and people living with CP [21]. As a preliminary step towards improved awareness and knowledge about chronic pain, a scale was then developed to measure knowledge, beliefs, and attitudes towards CP in the community [21]. This scale was meant to be more generic and to be used in the general population including HCP. The Chronic Pain Myth Scale (CPMS) was originally developed in French-Canadian. To the best of our knowledge, no scales are available to evaluate common societal misconceptions towards CP in English. Considering the increase in ED frequentations by people suffering from CP and the risks of undertreatment due to stigmatization in this clinical setting, this study thus aimed at determining to what extent a new English adaptation of the CPMS could provide valid scores among US emergency nurses. The internal consistency, construct validity, and internal structure of the translated scale were thus examined.

## 2. Methods

### 2.1. The French-Canadian CPMS

The original instrument includes 26 items and was found to measure three dimensions: knowledge, beliefs, and attitudes towards (1) people suffering from CP, (2) biopsychosocial impacts of CP, and (3) treatment of CP [21]. Items are scored on a 5-point Likert scale ranging from 1 (completely disagree) to 5 (completely agree). Negatively formulated items (2, 3, 4, 5, 7, 8, 9, 14, 20, 21, 24, 25, and 26) need to be reversed, so a higher score reflects better knowledge, attitudes, and beliefs. Total scores are calculated for each of the three subscales. Among French-speaking individuals from the general population, internal consistency of the CPMS was achieved for all three subscales (*α* = 0.72–0.82), in addition to its construct validity [21].

### 2.2. Adaptation and Translation Process

#### 2.2.1. Step 1: English Translation and Adaptation

The first step was the forward translation of all items, which was conducted by one of the bilingual investigators (AL) from French-Canadian to English. An adaptation of the translated version by a unilingual English-speaking nurse (MK) without specific expertise regarding chronic pain was done. Some suggestions were made such as using “really hurt” instead of “really have pain” or “may worsen” instead of “may aggravate.” Some other modifications were related to the context. For instance, the French-Canadian version included the description of winter sports such as snowmobile; it was then proposed to replace it by motorized sports to be more generic to all sorts of North American areas.

#### 2.2.2. Step 2: Synthesis Meeting

A synthesis meeting was then held using a web-based screen-sharing application to reconcile discrepancies and agree on a preliminary English version.

#### 2.2.3. Step 3: Backward Translation

The third step consisted in the backward translation of the English items in French-Canadian by one of the bilingual investigators (GM) and without consulting the original version.

#### 2.2.4. Step 4: Review Committee

The fourth step consisted in the reviewing of the different versions by the two co-investigators (AL and GM). A chart reporting the original French-Canadian version, the translated and adapted English version, the backward French-Canadian translation, and comments from Steps 1, 2, and 3 were used. Consensus was reached after any discrepancies were resolved.

#### 2.2.5. Step 5: Pretest

Guidelines recommend that the prefinal version of a questionnaire should be pretested among 30–40 people from the target setting [22]. Therefore, the prefinal translated version of the CPMS was pilot-tested in a convenience sample of 30 graduate nursing students. In July 2017, after obtaining ethical approval, the pilot study was advertised through the Florida State University College of Nursing's website and through the graduate nursing students' mailing list, where a link to an online survey (Qualtrics®) was posted. Once they clicked on the link, they were taken to the first page of the survey where information was given about the study to ensure informed consent and where it was underlined that by taking the survey, they were giving their consent to participate. The instructions included to complete the questionnaire and to annotate any suggestions regarding the clarity. Each of the 26 items was accompanied by a check box (clear vs needs improvement) and a text box where they could include comments. No further modifications were necessary as only 2 participants made some minor comments. The final version of the English CPMS is presented in Table 1.

### 2.3. Validation Study Design and Setting

The validity of the English CPMS was evaluated through a web-based cross-sectional study. A voluntary, convenience sample was used. Inclusion criteria included nurses who have worked in an ED for at least 6 months, who can read English, have access to the internet, and have the ability to fill out an electronic-based survey. A definition of CP as pain being present daily or repeatedly for more than 3 months was provided at the beginning of the survey.

The recruitment process included a link to the survey on the Emergency Nurses Association's (ENA) website that was located under the External Research Opportunities tab. No additional information on the study was provided on the ENA website. Facebook, a popular social media platform, was used to recruit participants from the different ENA's state chapters. A participant information clip of 35 seconds featuring a nurse (MK) explaining the focus of the study and eligibility criteria was posted. A link to the survey was provided at the end of the PIC. By clicking on the link, potential participants were taken to the introduction page where information about the study was provided to ensure informed consent. Before beginning the questionnaire, they were informed that by taking the survey they were agreeing to participate and giving their consent for the use of their responses. The study was approved through the Florida States University's Institutional Review Board.

Data were collected using a Qualtrics® electronic survey which allows the direct transfer of data into an SPSS database. The survey was approximately 20 minutes in length to complete and was available from October 1, 2017, to November 1, 2017. The following sociodemographic characteristics were collected at the beginning of the survey: age, sex, ethnicity, highest degree, clinical role, current setting of practice, years of experience, and suffering from chronic pain, i.e., pain present daily or repeatedly for more than 3 months (y/n). Upon completion of the survey, participants were provided with the option to enter a drawing for one of the four 25$ Visa gift cards by providing an email address. Responses were limited to one IP address, and a screening for duplicates was done during the database cleaning process.

### 2.4. Data Analysis

Sociodemographic characteristics (i.e., age, sex, years of experience, clinical setting, and role) and CPMS scores were analyzed descriptively. Frequency tables, means, and standard deviations were used to summarize data for each subscale. Reliability was assessed via internal consistency of the three CPMS subscales reflected by unstandardized Cronbach's alpha coefficients. These coefficients range between 0 (weak reliability) and 1 (perfect reliability) with a cutoff >0.7, usually reflecting adequate internal consistency/reliability [23]. Floor or ceiling effects were considered present if more than 15% of participants achieved the lowest or highest scores in each of the subscales [24]. Convergent construct validity can be determined by comparing scores between groups that are expected to score higher or lower on the scale (known-groups or extreme-groups technique) [25]. As it was done to validate the original French-Canadian instrument, the following subgroups were compared using a *T*-test: nurses suffering versus nurses not suffering from CP (i.e., CP > 3 months). It was hypothesized that nurses who suffer from chronic pain would score higher in each subscale than nurses who do not. Regarding the internal structure, since the original questionnaire is at an early stage of development and this is the first iteration of the translated tool, confirmatory factor analysis seemed premature and restrictive [26]. Hence, exploratory factor analysis was performed as it would allow to identify items that might be problematic. The factors were extracted using principal axis factoring, and orthogonal varimax rotation was used in order to remain consistent with the previous study. According to the literature, loadings greater than 0.32 can be considered acceptable in a sample of at least 300 [27]. There are no general rules to determine the required sample size for validation studies. However, rules of thumb (e.g., 3–10 participants for each item contained in the scale to be validated) and absolute minimum sample size can be used for specific tests such as factor analysis [25, 28–30]. It was then determined that at least 260 participants (10 participants × 26 items) were required to validate the internal structure of the CPMS. Data were analyzed using IBM SPSS version 25.

## 3. Results

### 3.1. Participants' Characteristics

A total of 571 participants were recruited among whom 482 completed the CPMS. The sample comprised primarily females (90.9%), Caucasians (90.2%), and Bachelor's prepared nurses (55.6%). Most nurses worked in an emergency department (92.3%). Sixty-seven percent of the sample reported that they were not suffering from chronic pain (i.e., pain present daily or repeatedly for more than 3 months). Characteristics of the sample are presented in Table 2.

### 3.2. Internal Consistency

As shown in Table 3, Cronbach's *α* coefficients reached the 0.7 cutoff, except for the second subscale (knowledge, beliefs, and attitudes towards biopsychosocial impact of CP). Moreover, internal consistency was maintained on the first subscale among nurses suffering from CP (*α*=0.88) and nurses not suffering from CP (*α*=0.88), the same was true for the third subscale (*α*=0.71 and *α*=0.70). For the second subscale, Cronbach's alpha was calculated as 0.67 with *α*=0.64 for nurses suffering from CP and *α*=0.68 for nurses not suffering from CP. No floor or ceiling effects were found to be present in the data for any of the three subscales.

### 3.3. Construct Validity of the English CPMS

Statistically significant differences (*p* < 0.05) were found between subgroups for the three subscales. Nurses who suffer from CP showed better knowledge, beliefs, and more positive attitudes regarding people suffering from CP (*M* = 32.70, SD = 6.22) than nurses who did not suffer from CP (*M* = 30.36, SD = 5.12; *p* < 0.001). Additionally, nurses who suffer from CP had better knowledge, beliefs, and more positive attitudes regarding biopsychosocial impacts of CP (*M* = 40.10, SD = 3.92) than nurses who did not suffer from CP (*M* = 39.26, SD = 3.87; *p*=0.025). However, nurses who suffer from CP had lower knowledge, beliefs, and less positive attitudes regarding treatment of CP (*M* = 26.63, SD = 3.95) than nurses who did not suffer from CP (*M* = 28.02, SD = 3.19; *p* < 0.001).

### 3.4. Internal Structure of the English CPMS

**Figure 1:**
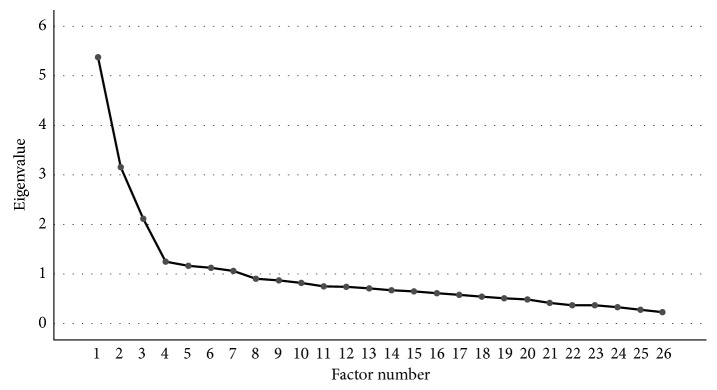
Scree plot for the exploratory factor analysis. The scree plot indicates the retention of 3 factors.

The Keiser–Meryer–Olkin measure of sampling adequacy (KMO = 0.84) and Bartlett's test of sphericity (*p* value <0.001) both indicated that factor analysis was appropriate for our data [27]. Seven different factors had eigenvalues greater than Kaiser's criterion of 1. However, based on the scree plot (Figure 1), a reliable criterion for large samples [31], three factors were retained such as it was the case for the original French-Canadian CPMS. Three factors explaining up to 41% of the variance were then extracted. After rotation, the three factors explained 34.09% of the variance. As it was the case for the original CPMS, items 1 through 9 had the highest loadings for factor 1, items 10 through 19 had the highest loadings for factor 2, and items 20 through 26 had the highest loadings for factor 3 (see Table 1 for rotated factor structure). The majority of items (92.3%) showed no crossloadings (items >0.32 on two or more factors [32]).

## 4. Discussion

The CPMS is a generic tool to measure knowledge, attitudes, and beliefs towards people suffering from CP, the biopsychosocial impacts of CP, and its treatment that was never adapted in English nor tested specifically among nurses. This study aimed at examining preliminary validity of the English version of the CPMS according to some psychometric qualities in a sample of emergency nurses. Our study suggests that the English CPMS was reliable and valid among such a population.

Acceptable reliability (*α* > 0.7) for research purposes in large populations [23] was reported for the first and third subscales. The second subscale's reliability coefficient was below the cutoff (*α*=0.67) but is still considered adequate [33]. Moreover, whether nurses were suffering or not from CP did not interfere with reliability. After applying the known-groups technique for construct validity, better knowledge and more positive attitudes towards people suffering from CP and its biopsychosocial impacts were found among nurses suffering from CP (as expected). This finding converges with results from the validation studies of the French-Canadian CPMS [21] and other scales that compared pain and pain-free participants [34–36]. Interestingly, the only exception was for knowledge, beliefs, and attitudes regarding treatment of CP, where CP sufferers scored lower. This result was also found in the validation study of the French-Canadian CPMS in the general population [21]. A logical explanation could be related to the fact that people suffering from CP have trouble finding some relief as well as being believed regarding their pain experience. As a result, they feel discouraged which could lead to negative attitudes towards the treatment of CP. It is also noteworthy that nurses' scores on the first subscale (knowledge, beliefs, and attitudes towards people suffering from CP) were lower than scores in HCP in general (*M* = 31.10, SD = 5.64 vs *M* = 39.95, SD = 4.32) [21]. This observation corroborates the issue of stigmatization of CP patients from HCP [10, 11] and more specifically from ED nurses [5, 8, 12]. Lastly, similar internal structure was found supporting the 3-factorial nature of the original CPMS. Considering that four items of the second CPMS subscale presented factor loadings <0.32, their relevance could be questionable. However, removing these items from the CPMS did not change our conclusions regarding its internal consistency of construct validity. All 26 items were thus kept. Hence, the vast majority of items demonstrated robust loadings on their respective factors (>0.4 or >0.5 [32]), which provides preliminary support for the cross-cultural equivalence of the English CPMS [37].

### 4.1. Strengths and Limitations

To our knowledge, this is the first study to adapt and validate the use of the English CPMS. Before validating several psychometric properties, careful attention was given to the cross-cultural adaptation including a pretest with 30 participants [22]. Although our sample was mainly composed of Caucasian women, these demographics are representative of the nursing population in the US [38]. Moreover, the participants reported various levels of clinical experience and were from at least 20 different states across the US. Another strength of this study is the sample size. While no a priori sample size calculation was conducted, the sample was much larger than rules of thumb (*n*=78–260) [25].

Considering that psychometric properties are not fixed and can vary according to a given population [25], we cannot presume that these will be the same in a different sample and/or sample size. The English version of the CPMS should be validated in a heterogeneous sample from the general population, which could lead to confirmatory factor analysis and support of translation invariance [37]. Of note, the response rate could not be calculated, which keeps us from evaluating the risk of a nonresponse bias. Additionally, participants in the sample were self-selected which could lead to a participation bias.

## 5. Conclusion

Given that the intent in developing the CPMS was to offer a “one fits all” tool, it is encouraging to observe similar psychometrics in terms of reliability, construct validity, and internal structure in a sample composed solely of HCP. The English CPMS is a promising tool to measure knowledge, beliefs, and attitudes towards CP. Its adaptation and preliminary validation in a sample of nurses is a first step towards the improvement of chronic pain management. Indeed, this tool could be used to measure educational needs, design awareness, and educational programs, as well as evaluating their outcomes.

## Figures and Tables

**Table 1:** Rotated factor matrix of the English CPMS (*n*=482).

Items	Factor 1	Factor 2	Factor 3
(1) Really have pain, it is not in their head	**0.409**	0.263	0.056
(2) Just want to be prescribed drugs	**0.717**	0.081	0.008
(3) Try to obtain sick leave to stop working	**0.746**	0.006	−0.091
(4) Just want to be lazy and not accomplish their daily tasks	**0.716**	0.203	0.064
(5) Complain of pain to get attention from others	**0.778**	0.089	−0.011
(6) Really want to get better	**0.594**	0.236	−0.041
(7) Complain about their pain, but continue their activities (e.g., sports, motorized sports, and watercraft). Their pain should not be that bad	**0.543**	0.095	0.008
(8) Become dependent on their medications, like drug addicts	**0.467**	0.028	−0.200
(9) Often tend to exaggerate the severity of their condition	**0.745**	0.144	−0.077
(10) Chronic pain causes several physical symptoms (e.g., muscle tension, change in appetite, reduced mobility, and fatigue)	0.220	**0.515**	0.122
(11) Chronic pain can have a direct impact on sex life	0.262	**0.651**	0.025
(12) People with chronic pain are sometimes not accepted by their relatives	0.126	**0.494**	−0.026
(13) Chronic pain may be associated with negative emotions (e.g., fear, anger, or sadness)	0.184	**0.591**	0.150
(14) People with chronic pain do not tend to isolate themselves	0.208	**0.281**	0.072
(15) People with chronic pain usually have more difficulty resisting stressful events of daily life	−0.128	**0.274**	0.083
(16) The risk of death by suicide is higher among people with chronic pain than in the general population	0.144	**0.500**	−0.015
(17) Chronic pain costs billions of dollars to our society	−0.247	**0.270**	0.209
(18) People with chronic pain do not always have access to healthcare services to treat their condition	0.190	**0.356**	−0.125
(19) Doctors lack time to treat chronic pain	−0.011	**0.265**	0.005
(20) Consulting a psychologist is useless unless the person with chronic pain is depressed	−0.048	0.129	**0.443**
(21) There is not much to do to improve chronic pain	0.113	0.060	**0.388**
(22) Good sleeping habits help reduce chronic pain	−0.185	0.286	**0.526**
(23) A balanced diet helps reduce chronic pain	−0.193	0.271	**0.616**
(24) Doing physical exercise may worsen chronic pain	−0.003	−0.175	**0.700**
(25) Working may worsen chronic pain	−0.132	−0.255	**0.640**
(26) The treatment of chronic pain is in the hands of healthcare professionals and not those of the patient	0.040	0.010	**0.350**
Eigenvalues before rotation	5.38	3.16	2.12
Percentage of variance explained after rotation	16.26	9.38	8.45

Extraction method: principal axis factoring. Rotation method: varimax with Kaiser normalization. Bold type indicates factor loading for each item.

**Table 2:** Sample description.

Variable		*n* (482)	%
Sex	Male	43	8.9
	Female	438	91.1

Ethnicity	Caucasian	435	91.0
	African American	10	2.1
	Latino/Hispanic	14	2.9
	Asian	3	0.6
	Native American	4	0.8
	Other	12	2.5

Age	18–35	207	43
	36–55	219	45.5
	56–75	55	11.5

Highest level of education completed	Professional degree	133	27.7
	Bachelor	267	55.6
	Masters	50	10.4
	Doctorate	5	1
	Other	25	5.2

Clinical role	Registered nurse	444	92.3
	Clinical nurse specialist	4	0.8
	Nurse practitioner	20	4.2
	Other	13	2.7

Current clinical practice	Inpatient/acute care	17	3.5
	Outpatient	4	0.8
	Primary care	3	0.6
	Emergency department	445	92.5
	Other	12	2.5

Years of experience	0–5	164	34.1
	6–10	117	24.3
	11+	200	41.6

Suffer from chronic pain	Yes	158	32.8
	No	323	67.2

**Table 3:** Cronbach's alpha coefficients and descriptive statistics of the English CPMS.

	Subscale 1	Subscale 2	Subscale 3
Cronbach's *α*	0.86	0.67	0.72
Mean score ± SD	31.10 ± 5.64	39.55 ± 3.91	27.53 ± 3.56
Median (range)	31 (9–45)	39 (23–50)	28 (15–35)
Possible scores	9–45	10–50	7–35

## Data Availability

The data used to support the findings of this study are available from the corresponding author upon request and approval from the Florida State University's Institutional Review Board.
